# Supplementary material for: The genome sequence of the Sandhill Knot-horn moth, Anerastia lotella Hübner, 1813
Source: Wellcome Open Res. 2025 Mar 19;10:148. [Version 1] doi: 10.12688/wellcomeopenres.23898.1 (PMC12485358; doi:10.12688/wellcomeopenres.23898.1)
Supplement: Peer review history

# Reviewer report: Reviewer response for version 1

**DOI:** 10.21956/wellcomeopenres.26365.r122431

**Published:** 2025-05-09

**Authors:** Shiqi Luo

**Affiliations:** 1 China Agricultural University, Beijing, China

This study presents the genomic information of the Sandhill Knot-horn moth *Anerastia lotella*. The assembly quality is high. The authors also provide essential information and data storage details in Table 1 and 2, and specifies the software used in Table 4. A suggested improvement would be to provide more specific parameter details for the software listed in Table 4.

Are sufficient details of methods and materials provided to allow replication by others?

Yes

Is the rationale for creating the dataset(s) clearly described?

Yes

Are the datasets clearly presented in a useable and accessible format?

Yes

Are the protocols appropriate and is the work technically sound?

Yes

Reviewer Expertise:

insect functional and evolutionary genomics

I confirm that I have read this submission and believe that I have an appropriate level of expertise to confirm that it is of an acceptable scientific standard.

# Reviewer report: Reviewer response for version 1

**DOI:** 10.21956/wellcomeopenres.26365.r120978

**Published:** 2025-03-25

**Authors:** Andrew Mongue

**Affiliations:** 1 Department of Entomology and Nematology, University of Florida, Gainesville, Florida, USA

As expected of the Tree of Life Project, the genome assembled here is high-quality and the data are readily available. 

 The background on natural history is rather brief, but I appreciate that there may be less ecological information for some species than others. One thing perhaps worth mentioning is the relatively large genome size recovered here. I am not a Pyralid specialist but I was immediately struck by how much bigger the Anerastia lotella genome is than say the Plodia interpunctella genome (<300Mb). Are there enough pyralid sequences yet to get a sense of how genome size evolves across this family?

Are sufficient details of methods and materials provided to allow replication by others?

Yes

Is the rationale for creating the dataset(s) clearly described?

Yes

Are the datasets clearly presented in a useable and accessible format?

Yes

Are the protocols appropriate and is the work technically sound?

Yes

Reviewer Expertise:

invertebrate genomics, Lepidoptera, sex chromosomes, population genetics

I confirm that I have read this submission and believe that I have an appropriate level of expertise to confirm that it is of an acceptable scientific standard.
